# *Blastocystis* Isolates from Patients with Irritable Bowel Syndrome and from Asymptomatic Carriers Exhibit Similar Parasitological Loads, but Significantly Different Generation Times and Genetic Variability across Multiple Subtypes

**DOI:** 10.1371/journal.pone.0124006

**Published:** 2015-04-29

**Authors:** Gie-Bele Vargas-Sanchez, Mirza Romero-Valdovinos, Celedonio Ramirez-Guerrero, Ines Vargas-Hernandez, Maria Elena Ramirez-Miranda, Joel Martinez-Ocaña, Alicia Valadez, Cecilia Ximenez, Eduardo Lopez-Escamilla, Maria Elena Hernandez-Campos, Guiehdani Villalobos, Fernando Martinez-Hernandez, Pablo Maravilla

**Affiliations:** 1 Escuela Superior de Medicina, Instituto Politecnico Nacional, Plan de San Luis y Diaz Mirón s/n, Casco de Santo Tomas, 11340, Mexico City, DF, Mexico; 2 Hospital General “Dr. Manuel Gea Gonzalez”, Calzada de Tlalpan 4800, Seccion XVI, 14080, Mexico City, DF, Mexico; 3 Hospital Infantil de Mexico Federico Gomez, Dr. Marquez 162, Doctores, 06720, Mexico City, DF, Mexico; 4 Centro Especializado de Atención Primaria “Melchor-Ocampo, Bicentenario”, Secretaria de Salud del Estado Mexico, Estado de Mexico, 54880, Mexico; 5 Departamento de Medicina Experimental, Facultad de Medicina, Universidad Nacional Autonoma de Mexico, 04510, Mexico City, DF, Mexico; 6 Departamento de Ecologia Evolutiva, Instituto de Ecologia, Universidad Nacional Autonoma de Mexico, Avenida Universidad 3000, 04510, Mexico City, DF, Mexico; Instituto de Diagnostico y Referencia Epidemiologicos, MEXICO

## Abstract

*Blastocystis* spp is a common intestinal parasite of humans and animals that has been associated to the etiology of irritable bowel syndrome (IBS); however, some studies have not found this association. Furthermore, many biological features of *Blastocystis* are little known. The objective of present study was to assess the generation times of *Blastocystis* cultures, from IBS patients and from asymptomatic carriers. A total of 100 isolates were obtained from 50 IBS patients and from 50 asymptomatic carriers. Up to 50 mg of feces from each participant were cultured in Barret’s and in Pavlova’s media during 48 h. Initial and final parasitological load were measured by microscopy and by quantitative PCR. Amplicons were purified, sequenced and submitted to GenBank; sequences were analysed for genetic diversity and a Bayesian inference allowed identifying genetic subtypes (ST). Generation times for *Blastocystis* isolates in both media, based on microscopic measures and molecular assays, were calculated. The clinical symptoms of IBS patients and distribution of *Blastocystis* ST 1, 2 and 3 in both groups was comparable to previous reports. Interestingly, the group of cases showed scarce mean nucleotide diversity (π) as compared to the control group (0.011±0.016 and 0.118±0.177, respectively), whilst high gene flow and small genetic differentiation indexes between different ST were found. Besides, Tajima’s D test showed negative values for ST1-ST3. No statistical differences regarding parasitological load between cases and controls in both media, as searched by microscopy and by qPCR, were detected except that parasites grew faster in Barret’s than in Pavlova’s medium. Interestingly, slow growth of isolates recovered from cases in comparison to those of controls was observed (*p*<0.05). We propose that generation times of *Blastocystis* might be easily affected by intestinal environmental changes due to IBS probably because virulent strains with slow growth may be selected, reducing their genetic variability.

## Introduction

Irritable bowel syndrome (IBS) is defined as a functional bowel disorder in which abdominal pain or discomfort is associated with disordered defecation or with a change in bowel habits in the absence of an organic cause [1–2]. The pathophysiology of IBS remains elusive and no mechanism is unique to, or characteristic of, IBS. There are probably several interconnected factors which occur to varying degrees in patients that account for the clinical symptoms of IBS, these include altered gut reactivity (colonic and/or small bowel motility) in response to luminal or psychological stimuli, hypersensitive viscera or gut, enhanced visceral perception and pain [3–4].

*Blastocystis* spp., an intestinal parasite, is one of the most common parasites worldwide in humans and although its ability to cause disease has been questioned, some reports have demonstrated that this microorganism is associated to the development of IBS [5–9]. Up to 17 subtypes (ST) have been described in this parasite, based on its small subunit rDNA analysis (SSUrDNA), humans are colonized mainly by ST1–ST4; their prevalence seems to vary from country to country and among communities within the same country. Some analyses have shown a lack of association between ST and IBS [10–12]. In addition, a relevant phenotypic variation in size, growth and clumping was documented among ST3 isolates from patients with gastrointestinal alterations and in asymptomatic carriers [13]. Many culture media, as well as physical and chemical parameters have been tested for *Blastocystis* growth; some of them generated improvement of its diagnosis [14–18], but no links between *in vitro* generation times indifferent *Blastocystis* ST, and their genetic variation, have been reported. Therefore, the objective of present study was to assess generation times and genetic polymorphism of *Blastocystis* cultures from IBS patients and from asymptomatic carriers.

## Materials and Methods

### Samples

Fecal samples of 50 IBS patients (cases) and 50 unrelated healthy volunteers (controls) with *Blastocystis* as a unique infection, diagnosed by flotation-concentration Ferreira’s technique were studied. For the control' group, all participants were recruited when attending the Blood Bank of the Hospital General “Dr. Manuel Gea Gonzalez” for a voluntary and altruistic blood donation; after a clinical check-up they were invited to participate in the present study by providing fecal samples for a coprological diagnosis. Cases were recruited during their medical consultations. Informed consent was obtained from each participant before their recruitment. IBS patients were diagnosed according to the Rome III criteria [2], which refer recurrent abdominal pain or uncomfortable sensation at least 3 days per month in the last 3 months associated with 2 or more of the following: i) improvement with defecation, ii) onset associated with a change in frequency of stool release, iii) onset associated with a change in form or appearance of stools; in order to discard the presence of Rotavirus and pathogenic enterobacteria, all samples were tested with a commercial Rota Test (CerTest, Spain) and VITEK-2 system (BioMérieux, France), respectively.

### Culture

Using templates of the Kato-Katz technique [19], 50 mg of feces of each participant were cultured in 2mL of Barret’s medium [20–21] and of Pavlova’s medium [16, 22]; both complemented with 10% horse serum, as well as Penicillin G (1,000IU/mL) and Streptomycin (100μg/mL). Sterile 2.5mL screw cap tubes were used for incubation at 37°C; all assays were performed in duplicate. Two aliquots of ~30 and 500μL were recovered at the beginning and at 48h. The first aliquot was used to measure the concentration of *Blastocystis* cells by Neubauer chamber, whilst the second was used to obtain DNA. At the end of the experiment, to facilitate counting parasites, cultures were fixed in 10% formalin.

### DNA extraction and quantitative PCR (qPCR) assays

Total DNA from each stool sample cultured was obtained using a conventional extraction protocol with proteinase K, phenol/chloroform solution and precipitated with isopropanol [23]; DNA extracts were stored at -20°C until molecular analysis. Absolute quantification was performed by qPCR in a Light Cycler480-II system (Roche, Germany), using as positive control a sample with a known number of parasites (8,920 *Blastocystis* organisms/mg feces) and a DNA concentration of 160ng/μL, determined by UV-spectrophotometry. A set of primers that amplify a region of the *Blastocystis* small subunit of ribosomal DNA (SSUrDNA) gene, previously reported by Poirier et al. [24] were used and the amplification was performed with SYBR green kit (Fermentas, CA), 3.5 mM MgCl_2_, 0.2μM of each primer and 200ng of DNA extracted. The amplification conditions were: an initial denaturation cycle of 95°C for 10min, 45 amplification cycles 95°C 20s, 65°C 20s and 72°C 20s. To establish the coefficient of correlation of the qPCR assays, 10 fold dilutions of DNA control were used in duplicated experiments. For data analysis, the melting curves at 95°C 5s and of 65°C to 97°C for 1min was performed; afterwards a concentration curve was obtained and all DNA samples were amplified and compared with this control.

### *Blastocystis* subtype (ST) determination and genetic variation analysis

High Resolution Melting (HRM) analysis was conducted in order to support the ST identification, in some DNA samples that were amplified in 96-well plates, and HRM curves acquisition was performed, similarly to qPCR, under the same amplification conditions and system, but using a Light Cycler HRM master kit (Roche, Germany) to assign a melting temperature. DNA samples with ST identified by sequencing (ST1, ST2, ST3 and ST7) were included as references for wild-type curves. According to Wittwer et al. [25] and Gonzalez-Bosquet et al. [26], normalization and background subtraction were first performed by fitting an exponential to the background surrounding the HRM transitions of interest; the normalized HRM curves were temperature-overlaid, to eliminate slight temperature errors between wells or runs; after, different plots of these normalized and temperature-overlaid curves were obtained by deducting the fluorescence difference of each curve from the average wild-type curve at all temperature points. Thus, HRM profile with a plot interpreted by the software to be different from the averaged wild-type curve, were considered to be suspicious of harboring a nucleotide change, mutation or variant that, in our particular case, corresponded to each ST. Direct sequencing was performed to identify the ST in DNA samples. Direct qPCR product sequencing was performed according to Poirier et al [24], after purification with a QIAquick PCR Purification Kit (Qiagen, Germany). Sequencing was performed by a commercial supplier using the same primers as those used for qPCR (BL18SPPF1 and BL18SR2PP) that amplify a DNA fragment of 320 to 342bp, depending on the subtype.

All sequences were subjected to a BLAST search in the GenBank database; multiple alignments were performed using the CLUSTAL W [27] and Muscle [28] programmes with manual adjustment in MEGA 5.05 software [29]. Hasegawa Kishino Yano model with gamma distribution and invariable sites was the best fit model of nucleotide substitution for SSUrDNA, determined using the Akaike Information Criterion in Modeltest version 3.7 software [30]. The phylogenetic reconstruction using Bayesian inference was performed with the Mr. Bayes 3.1.2 program [31–33]. The analysis was done for 10 million generations with sampling trees every 100 generations. Trees with scores lower than those at the stationary phase (burn-in) were discarded, and the trees that reached the stationary phase were collected and used to build majority consensus trees. Other sequences were obtained from GenBank and used as subtype references. A genetic diversity analysis within and between populations was performed using DnaSPv4 [34] and included nucleotide diversity (π), haplotype polymorphism (θ), gene flow (*Nm*), genetic differentiation index (F_ST_) and Tajima’s D test. These indexes refer to the following: π, the average proportion of nucleotide differences between all possible pairs of sequences in the sample; θ, the proportion of nucleotide sites that are expected to be polymorphic in any suitable sample from this region of the genome. Both indexes are used to assess polymorphisms at the DNA level and to monitor diversity within or between ecological populations and examine the genetic variation in related species or their evolutionary relationships. F_ST_ is a typical genetic statistic used to measure differentiation between or among populations. The common used values for genetic differentiation are as follows: 0 to 0.05, small; 0.05 to 0.15, moderate; 0.15 to 0.25, great; values above 0.25 indicate huge genetic differentiation. The gene flow or migration index (*Nm*) refers to the movement of organisms among subpopulations; those strongly differentiated have an *Nm*<<1, whereas an *Nm*>4 behaves as a single panmictic unit [35].

### Generation time

The generation times of *Blastocystis* isolates in the two media, based on microscopic measures and molecular assays, were calculated according to Zhang et al. [17], with the following equation: *T*_*g*_ = *(T*_*2*_ *-T*_*1*_ *)/(*log_2_ *(n*_*2*_ */n*_*1*_ *))*, where *T*_*g*_ denotes the generation time, *n*_*1*_ represents the number of cultured parasitic organisms at the initial time (*T*_*1*_), and *n*_*2*_ represents the number of parasitic cells at subsequent time (*T*_*2*_). Thus, (*T*_*2*_ *-T*_*1*_) = 48 hours of *in vitro* culture.

Due to absolute quantification by qPCR, it was necessary to consider i) the size of the *Blastocystis* genome ~18.8Mbp [36]; ii) 1pg of DNA ~978Mbp [37] and iii) the concentration of DNA control was 160ng/μL; thus, by cross multiplications, the number of copies of the genetic marker amplified were estimated.

### Statistical analysis

Descriptive statistics are expressed as mean and standard deviation (SD). Analysis by Student's t test for unrelated and related samples and Mantel–Haenszel test were applied; odds ratio (OR) and 95% confidence intervals (95% CI) were also obtained. Data analysis was performed with SSPS software Version 15.0 (SPSS Institute, Chicago, IL).

### Ethics statement

All procedures are in accordance with the provisions of the Regulations of the General Health Law in the Field of Health Research in Mexico: Title II, Chapter II, from research communities; the Ethics and Research Committees of the General Hospital “Dr. Manuel Gea Gonzalez” approved the protocol with the reference number 12-87-2012, and written informed consent was obtained from each participant before recruitment.

## Results

**Fig 1:**
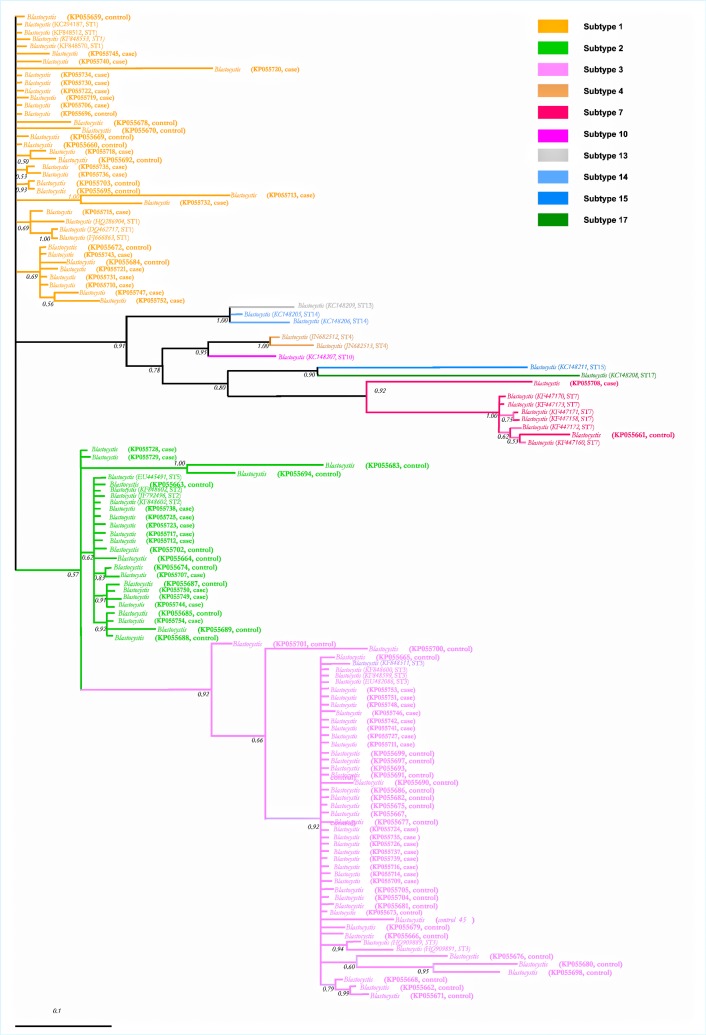
Phylogenetic inference of *Blastocystis* spp. Bayesian phylogenetic tree using a fragment of SSUrDNA sequences; the values of the nodes indicate posterior probabilities values using 10 million generations. GenBank accession numbers are included, as well as if correspond to a case or a control; each ST clade is shown in different branch colors.

Table 1 summarizes clinical symptoms and *Blastocystis* ST for cases and controls. All IBS patients presented abdominal pain and bloating, while flatulence and alternating diarrhea or constipation were the least frequent. All amplified DNA fragments were purified and sequenced; some DNA from the different ST (ST1, 2, 3 and 7) were amplified and melting analysis exhibited distinct temperature peaks, depending on ST; however, some of them were too close to distinguish among ST, therefore the sequence analysis leaded to identify the ST; 49 sequences were obtained for cases and 48 for controls (GenBank accession numbers KP055659-KP055754; however, sequence of the sample "control 45" was <200bp and its accession number was not assigned). ST1 was the most frequent in the cases' group (41%) followed by ST3 (33%); in contrast, for controls' group, the order of ST was inverted, because ST3 was the most frequent (54%) followed by ST1 (21%); distribution of ST2 and ST7 was similar between both groups. Interestingly, the group of cases showed a scarce mean nucleotide diversity and haplotype polymorphism as compared to control group (*p*<0.001), being ST2 the most diverse (Table 2). In contrast, ST1 was more variable in the cases' group. Regarding F_ST_ and *Nm* indexes, a small genetic differentiation and a high gene flow between different ST was found. Result of Tajima’s D test showed negative values for ST1-ST3. The phylogenetic tree (Fig 1) supported the grouping of our sequences in the ST1, 2, 3 and 7 clades.

**Table 1:** Characteristics of the carriers and of *Blastocystis* ST identified in IBS patients and in the control group.

	IBS group	Control group
**Age, years**	46±11.5	38±11.3
**Male/female, n**	11/39	10/40
**Clinical symptoms**	% (n)	% (n)
** Abdominal pain**	100 (50)	0 (50)
** Bloating**	100 (50)	0 (50)
** Flatulence**	45 (24)	0 (50)
** Diarrhea-Constipation**	40 (20)	0 (50)
***Blastocystis* ST**	% (n = 49)	% (n = 48)
** 1**	41 (20)	21 (10)
** 2**	24 (12)	23 (11)
** 3**	33 (16)	54 (26)^a^
** 7**	2 (1)	2 (1)

^a^ *p* = 0.043,

OR(95%IC) = 0.43(0.17–1.06).

**Table 2:** Population genetic indexes between different *Blastocystis* ST sequences.

*Blastocystis* ST	π		Θ		F_ST_	*Nm*	Tajima’s D (*p* value)
	IBS	Control	IBS	Control			
**1**	0.031	0.019	0.905	0.891	0.011	7.18	-2.256 (*p<*0.01)
**2**	0.002	0.324	0.682	0.972	0.039	6.48	-2.144 (*p<*0.05)
**3**	0.001	0.013	0.342	0.628	0.021	11.22	-2.562 (*p<*0.01)
**Mean±SD**	0.011±0.016	0.118±0.177	0.643±0.283	0.830±0.179			
**(*p*; 95%CI** ^**a**^ **)**	*p<*0.001; 95%CI = -0.16–0.06		*p<*0.001; 95%CI = -0.28–0.09				

^a^95%CI, 95% Confidence interval.

**Fig 2:**
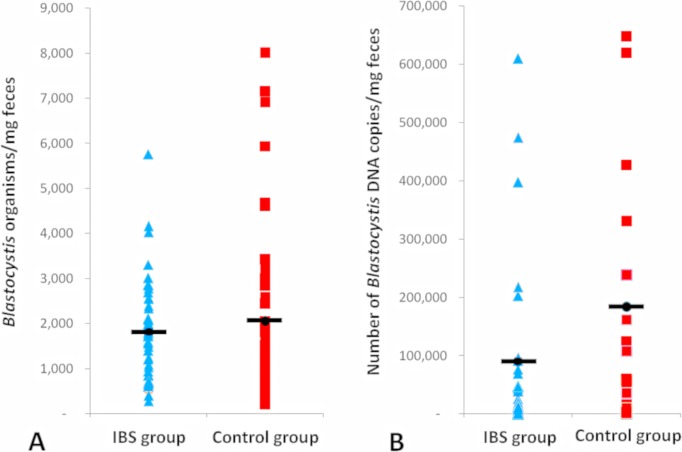
Parasitological loads estimated at the beginning of the study between cases and controls. Plots drew with all sample data according to the number of *Blastocystis* organisms/mg feces by microscopy (A) and number of *Blastocystis* DNA copies/mg feces by qPCR (B). The black bars mean the average in each group.

**Fig 3:**
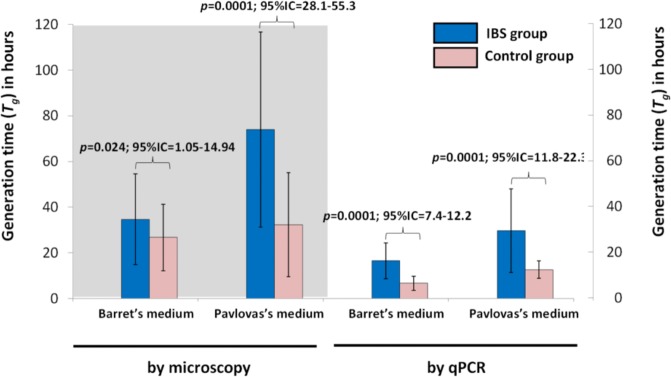
Generation time (*T*_*g*_) values of *Blastocystis* isolates between case and control groups. Average and standard deviation of *T*_***g***_ in Barret’s and Pavlova media, based on microscopic measures and qPCR assays.

During the counting of parasites in the cultured samples, the predominant stage was the vacuolar form in both media; after 48h of culture in both groups, few amoeboid and granular forms were observed, with a light increase of the granular form in Pavlova’s medium. No differences were found regarding the parasitological load between cases and controls in both media by microscopy and by qPCR, perhaps due to a high variance within data (Fig 2); however, some statistical differences between media and among groups were found, such as parasites showed faster growth in Barret’s medium than in Pavlova’s medium (p<0.001, t-test related samples). Interestingly, slow growth for isolates recovered from cases in comparison with controls was observed (*p*<0.05 by microscopy and *p* = 0.001 by qPCR; Fig 3).

## Discussion

Clinical symptoms in IBS patients infected with *Blastocystis* were concordant to those reported in Mexican populations [8, 38–40] and in other countries [6–7, 41–43]. Also, distribution of *Blastocystis* ST 1, 2 and 3 was comparable to other reports [8, 10, 38–40], showing no association with symptoms or the development of IBS, in contrast with those findings reported by Ramírez et al. [44], who found that all patients infected with *Blastocystis* ST2 presented diarrhea, while than asymptomatic carriers exhibited only ST1.

Recently, a study focused to assess the genetic variation and differentiation of *Blastocystis* subtypes recovered from symptomatic children from Michoacan, Mexico showed that by analysing partial sequences of the SSUrDNA the mean nucleotide diversity was π = 0.0179±0.0112 [40]; interestingly in the present work, a similar mean π = 0.011±0.016 for cases was calculated; in contrast, our control group showed a mean π almost ten times higher than the cases' group (π = 0.118±0.177), indicating that parasites from IBS patients exhibited less genetic variable than controls. Besides, F_ST_ and *Nm* indexes showed a small genetic differentiation and a high gene flow between cases and control groups for ST 1, 2 and 3, suggesting a large flow of parasites among carriers, regardless their ST. Besides, the negative values of Tajima’s D test advise a recent expansion process or an effect of purifying selection [35] that it might occur, particularly in those isolates from symptomatic *Blastocystis* carriers.

No statistical differences were observed in the parasitological load between cases and controls; besides, the HRM analyses did not allowed distinguishing unambiguously among ST, probably due to the relationship between the sensitivity of the technique and the high genetic polymorphism among ST [25]. The most significant findings in the present study were the dissimilarities in the *T*_*g*_ values from IBS patients and control groups, since a slow growth for isolates recovered from cases in comparison with controls was observed in the two media used; although Barret’s medium allowed a faster growth, measured by two different techniques, independently of their ST. Differences between *T*_*g*_ values obtained by microscopy and by qPCR, can be explained because both techniques measure dissimilar features and by the high sensitivity of the molecular assay, since with qPCR it has been possible to detect as few as 10^2^ *Blastocystis* parasites/g of stools as referred by Poirier et al. [24]. Tan et al. [45] studied some phenotypic characteristics of ten asymptomatic and ten symptomatic human-derived *Blastocystis* ST3 isolates, and found that asymptomatic isolates grew more rapidly (mean *T*_*g*_ = 9.3h) than symptomatic isolates (mean *T*_*g*_ = 15.3h) using Jones' medium supplemented with 10% horse serum. Recently, Ragavan et al. [13] studied 12 isolates of ST3 from four IBS patients, four gastrointestinal symptomatic patients and four asymptomatic carriers, finding that the asymptomatic isolates grew faster than symptomatic and IBS isolates which were similar with those reported by Tan et al. [45]; these data are in accordance with those of the present study. The media of Jones, Barret and Pavlova are simple sera-saline media; but the last one, phosphate salts and yeast extract are added to increase osmolarity [16, 20, 46]. In addition, Zhang et al. [17] evaluated the growth of *Blastocystis* in three commercially available liquid media (RPMI 1640, 199 Medium and Dulbecco’s modified Eagle’s medium) under a different pH, inoculum sizes and amounts of calf serum, finding that all culture factors modified the *T*_*g*_ in each case. Thus, we consider that *T*_*g*_ in *Blastocystis* is easily affected by intestinal environment changes (such as osmolarity, variations in the microbiota composition [47, 48], host diet, etc.) eventually leading to loss of infection, explaining the spontaneous remission observed by Sanchez-Aguillon et al. [49] during a cohort study.

Nutrient availability, an effective immune response and the host genetics can influence the response against pathogen challenges [50–52]; for example, it has been suggested that certain single nucleotide polymorphisms of interleukin-8 and -10 could change individual susceptibility increasing the relative risk in the development of IBS in *Blastocystis* carriers [53], Therefore differences for *T*_*g*_ observed in early cultures, free of the host immune response pressure and with sufficient nutrients, allow to assume differences in the cellular protein metabolism, not yet studied between isolates recovered from symptomatic and asymptomatic carriers. Besides, some cellular and physiological features, not codified by changes in the DNA sequence, such as DNA methylation, histone modifications and RNA-associated silencing, have been associated to virulence and evasion of the immune response in some protozoan parasites, such as *Entamoeba histolytica* and *Giardia lamblia* [54–56]. Thus, many aspects of the host-pathogen relationship in *Blastocystis* infections require to be studied, in order to clarify the pathogenic role of this microorganism.

Although it has been argued that genetic polymorphism inside each ST, could be related with the pathogenicity of *Blastocystis* [11, 57], little has been discussed about whether genetic differences in this microorganism may be associated with its growth, and if this might give some clue about its pathogenicity. In addition, it has been argued that some parasites exploit their hosts in a prudent way, taking the resources that they need without causing noticeable damage. Prudent exploitation yields sustainable benefits to the parasite as long as the host remains healthy. Other parasites attack their host more quickly and vigorously. Rapid exploitation may allow the parasites to achieve higher reproductive rates, but damage to the host reduces parasites' opportunity for sustainable yield. Following this economic line of thought, each parasite faces a trade-off when increasing the rate at which host resources are used; however, simple economic considerations will certainly not explain all aspects of parasitism and the severity of disease (virulence) [58]. The virulence trade-off hypothesis assumes that higher within-host replication rates lead to higher damage [59], however new opposite examples are emerging [60], i.e. Sistrom et al [61] analyzed the whole-genome sequences of *Trypanosoma brucei* complex from diverse hosts and regions, finding that a strong purifying selection associated to cytoskeleton structure and regulatory genes related with virulence; therefore, it is likely that carriers of *Blastocystis* virulent strains grow slowly (which would explain the chronic symptoms associated with this parasite) and by a purifying selection process, these strains could be directed towards specialization, showing reduced genetic variability. This idea is supported by our Tajima’s D test data and by a recent article, that it was focused in a comparative analysis of the population structure of eukaryotic pathogens using Multilocus Sequence Typing, finding that *Blastocystis* dataset corresponded to a population of recent origin having undergone a radiation process [62].
